# Facile fabrication of polyurethane-based graphene foam/lead zirconate titanate/polydimethylsiloxane composites with good damping performance†

**DOI:** 10.1039/c8ra00266e

**Published:** 2018-02-20

**Authors:** Chunmei Zhang, Yujie Chen, Hua Li, Wenchao Xue, Ran Tian, Roberto Dugnani, Hezhou Liu

**Affiliations:** State Key Laboratory of Metal Matrix Composites, School of Materials Science and Engineering, Shanghai Jiao Tong University Dongchuan Road No. 800 Shanghai 200240 China lih@sjtu.edu.cn; Collaborative Innovation Center for Advanced Ship and Deep-Sea Exploration, Shanghai Jiao Tong University China; Michigan-Shanghai Jiao Tong University Joint Institute China

## Abstract

In modern society, much more noise and vibration are produced in traffic and industrial systems, which is harmful to human health, equipment safety and the environment, therefore damping materials are becoming increasingly important. A piezoelectric damping composite could broaden the damping temperature range and enhance the damping loss factor simultaneously by introducing a dissipation route of mechanical to electrical to heat energy. In this paper, a novel piezo-damping polyurethane-based graphene foam (PGF)/PZT/PDMS composite (PGPP) was facilely fabricated using a one-step vacuum-assisted filling method. Using three-dimensional graphene foam as a conductive phase, and due to its three-dimensional network structure, the PGPP composite can reach the percolation threshold with a dramatically reduced amount of RGO sheets. The effects of PZT content and frequency on the damping properties of the PGPP composites were investigated, and the results show that the storage modulus, loss modulus and loss factor of the PGPPs are all greatly enhanced compared to those of the PDMS matrix. Due to their flexibility, the PGPP composites can be used as good surface coating damping materials over a wide temperature range at different frequencies.

## Abstract 2

Using three-dimensional graphene foam as the conductive phase, the temperature range where tan *δ* > 0.3 for a piezo-damping PGPP composite is broadened to −70 to 100 °C.

## Graphical abstract

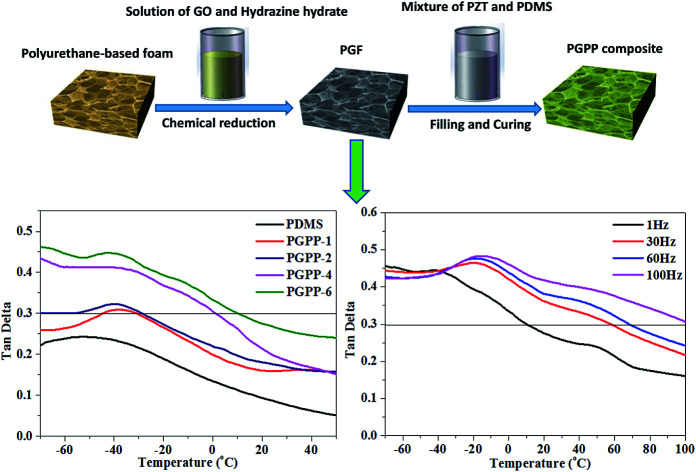

## 1. Introduction

Due to the rapid development of technology and economy, more and more noise and vibration, which are harmful to human health, industrial safety and the environment, are generated from activities such as traffic, construction and industrial production.^1–3^ Moreover, reducing noise and vibration is also important to guarantee military safety and the accurate operation of precision instruments. In recent years, the techniques used to reduce vibration and noise have attracted much more attention in modern engineering fields.^4,5^ Among these, polymers are the most commonly used damping materials due to the fact that the macromolecular segments of polymers begin to move near *T*_g_, and the friction between the segments can dissipate most external mechanical energy as heat.^6,7^ The loss factor (tan *δ*) is usually used to characterize the damping performance of a material and higher tan *δ* values represent better energy dissipation capacity. Generally, the requirement for the damping loss factor of practical engineering materials is for it to be above 0.3 and the temperature range where tan *δ* > 0.3 should be as wide as possible.^8–10^

As described above, polymers can be used as high performance damping materials due to their excellent viscoelasticity and good processibility. However, the good damping behavior of polymers is normally limited to a narrow temperature range of *T*_g_ ± 10 °C, which limits their practical use under many conditions. By blending different polymers with a desired *T*_g_ or by interpenetrating different polymer networks (IPNs), a broader glass transition temperature range can be obtained. Some IPNs like polyurethane/polystyrene, polyurethane/epoxy and unsaturated polyester/epoxy have been fabricated and the results showed that they exhibit good damping performance over a wide temperature range.^11–15^ However, the disadvantage was that the width and height of the loss factor peak could not be independently adjusted, as the broadening of the loss peak usually resulted in a decrease in its peak value.

The piezo-damping effect is used in new methods to improve the damping properties of materials. In recent years, various piezo-damping composites have been studied and some promising results have been obtained.^16–20^ Briefly speaking, a piezo-damping material is composed of a piezoelectric phase, a conductive phase and a polymer matrix. External mechanical energy, like vibration and noise, can be transformed into electrical energy through the piezoelectric effect of the piezoelectric ceramics, and then the generated electrical energy can be dissipated as heat energy as it flows through the composite’s resistive phase. To guarantee that the generated electrical energy has fully dissipated, the volume resistivity of the material should be adjusted to be in the semiconductor range.^21,22^ Sumita *et al.* compared the damping performance of the composites PZT/carbon black (CB)/PVDF and PLZT/CB/PVDF, and the results showed that the composite PLZT/CB/PVDF, which had piezoelectric ceramics with a higher electromechanical coupling factor, exhibited better damping behavior.^23^ Hori *et al.* fabricated a PZT/CB/epoxy resin (EP) composite and found that a maximum damping loss factor of 0.08 could be obtained with a CB content of 0.51 wt%, compared with a value of 0.035 for the EP matrix at room temperature. This demonstrated that the peak damping value was obtained at the percolation threshold, at which the CB particles electrically just came into contact with each other to form a conduction path.^21^ Tian *et al.* synthesized a PZT/multi-walled carbon nanotube (CNT)/epoxy composite, which showed a maximum damping value of about 0.22 at room temperature with a composition of 80 g PZT/1.5 g CNT/100 g epoxy.^22^ Wang *et al.* fabricated a PMN/CB/chlorobutyl rubber composite, which showed a maximum loss factor of 0.98 and a temperature range where tan *δ* > 0.5 from −52.8 to 3.0 °C at 25 wt% CB content.^24^ Liu *et al.* studied the damping properties of a PZT/CB/chlorobutyl rubber (CIIR)/poly(ethyl acrylate) (PEA) composite, and it was found that when the amounts of CB and PZT were between 10 and 30 vol%, with a volume resistivity between 10^5^ and 10^9.5^ Ω cm, a good damping performance can be achieved.^25^ Compared to polymers, piezo-damping composites exhibit less dependence on temperature and frequency. Moreover, the addition of high modulus piezoelectric and conductive fillers can enhance the mechanical behavior of the polymer matrix. However, for the piezo-damping composites described above, the amount of conductive fillers used are generally high, which is not only costly, but could also exert a negative effect on the mechanical properties of the polymer matrix.

In this paper, a piezo-damping composite PGPP was facilely fabricated by filling conductive polyurethane-based graphene foam with a PZT/PDMS mixture. Using a three-dimensional conductive network, the material can be adjusted to the percolation threshold with only a small amount of RGO, and meanwhile it can easily guarantee the uniform distribution of RGO sheets in the polymer matrix. The influence of frequency and PZT ceramic content on the dynamic mechanical properties of PGPPs was investigated, and the results and explanations are described in this paper.

## 2. Experimental

### 2.1 Materials

The polyurethane foams were purchased from Sichuan Hongchang Plastics Industrial Co., Ltd., China (the brand name is “Maryya”) and used without additional processing. Hydrazine hydrate (H_4_N_2_·*x*H_2_O, AR, ≥85.0%), sodium nitrate (NaNO_3_, AR, ≥99.0%), potassium permanganate (KMnO_4_, AR, ≥99.5%), concentrated sulfuric acid (H_2_SO_4_, AR, 95.0–98.0%), hydrochloric acid (HCl, AR, 36.0–38.0%), hydrogen peroxide (H_2_O_2_, AR, ≥30.0%) and ethyl acetate (AR, ≥99.5%) were purchased from Sinapharm Chemical Reagent Co., Ltd., China and all of the chemicals were used without further purification. Graphite powder was bought from Qingdao Huatai Graphite Co., Ltd., China. Graphene oxide (GO) was prepared using a modified Hummers’ method.^26^ The PZT ceramics were purchased from ZiBo Bailing Functional Ceramics Co., Ltd., China and the value of their piezoelectric coefficient (*d*_33_) before balling into powders was approximately 650 pC N^−1^. The PDMS used was Sylgard 184 from Dow Corning Corporation.

### 2.2 Preparation of polyurethane-based graphene foam (PGF)

**Fig. 1:**
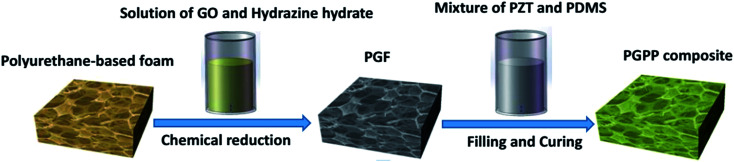
Schematic of the fabrication process of the PGF/PZT/PDMS composite (PGPP).

A schematic of the PGF fabrication process is shown in Fig. 1. Typically, 15 mg GO was dispersed into 30 ml water (*i.e.*, a concentration of 0.5 mg ml^−1^) and sonicated for 30 min at room temperature to obtain a clear solution. Hydrazine hydrate (38 μl) was added to the GO solution and stirred for 15 min until it was uniformly mixed with the GO. A commercial PU foam (approximately 30 cm^3^ in volume) was put into the solution, and after a repeated squeezing and vacuum degassing procedure, the composite was transferred into a 50 ml Teflon vessel. Subsequently, the vessel was sealed and placed in an oven heated to 95 °C for 12 h. The resulting monolithic gel-like product was taken out and washed with ethanol and deionized water several times to remove impurities. After drying in an oven at 60 °C for 12 h, the sample PGF-0.5 was obtained. The amount of hydrazine hydrate used was always 2.5 times the amount of GO by weight. Changing the GO mass to 3 mg and 30 mg (GO concentration of 0.1 and 1 mg ml^−1^) gave PGF samples that were named PGF-0.1 and PGF-1, respectively.

### 2.3 Fabrication of PGPP composite

The composite PGPP was facilely fabricated using a one-step vacuum-assisted filling method. As shown in Fig. 1, typically, a certain amount of PDMS and crosslinking agent was dissolved in ethyl acetate and stirred for several minutes to get a clear solution. Then a certain amount of PZT powder was added and the mixture was stirred vigorously for about 2 hours to make the PZT ceramics disperse uniformly in the PDMS matrix. Subsequently, the mixture was poured into the prepared PGF and placed under vacuum for about 60 minutes to remove the ethyl acetate solvent and the bubbles trapped in the composite. Afterwards the composite was cured at 120 °C for 2 h to finally obtain PGPPs. The composite PGPPs with different amounts of PZT ceramics were named PGPP-1, PGPP-2, PGPP-4 and PGPP-6 when changing the mass ratio of PZT and PDMS to 1 : 1, 2 : 1, 4 : 1 and 6 : 1, respectively. The mass ratio of PDMS to the crosslinking agent in the experiment was held constant at 10 : 1.

### 2.4 Characterization and testing

X-ray diffraction (XRD) spectra were acquired using a D/MAX2550/PC spectrometer, using Cu Kα radiation from 8° to 80° at a scan rate of 5° min^−1^ at 35 kV and 200 mA. Scanning electron microscope (SEM) images were obtained on a Hitachi S-4800 field-emission SEM operated at 10 kV. X-ray photoelectron spectroscopy (XPS) was performed using a Kratos Axis Ultra DLD spectrometer. Raman spectra were taken on a SENTERRA R200 Raman spectrometer with 532 nm laser excitation. Volume resistivity was measured using a ZC-36 high resistance meter from the Sixth Electric Meter Factory of Shanghai. A quasistatic *d*_33_ piezometer (model/ZJ-3A, China) was used to measure the piezoelectric coefficient of the composite. Dynamic mechanical measurements were taken on a Perkin-Elmer DMA 8000 instrument and rectangular specimens of 10 × 8 × 1.2 mm were used for the tests. The material properties were measured in compression mode at multifrequency (1, 30, 60 and 100 Hz) with a temperature range of −70 to 100 °C at a heating rate of 5 °C min^−1^ and the storage modulus, loss modulus, and loss factor were obtained simultaneously.

## 3. Results and discussion

**Fig. 2:**
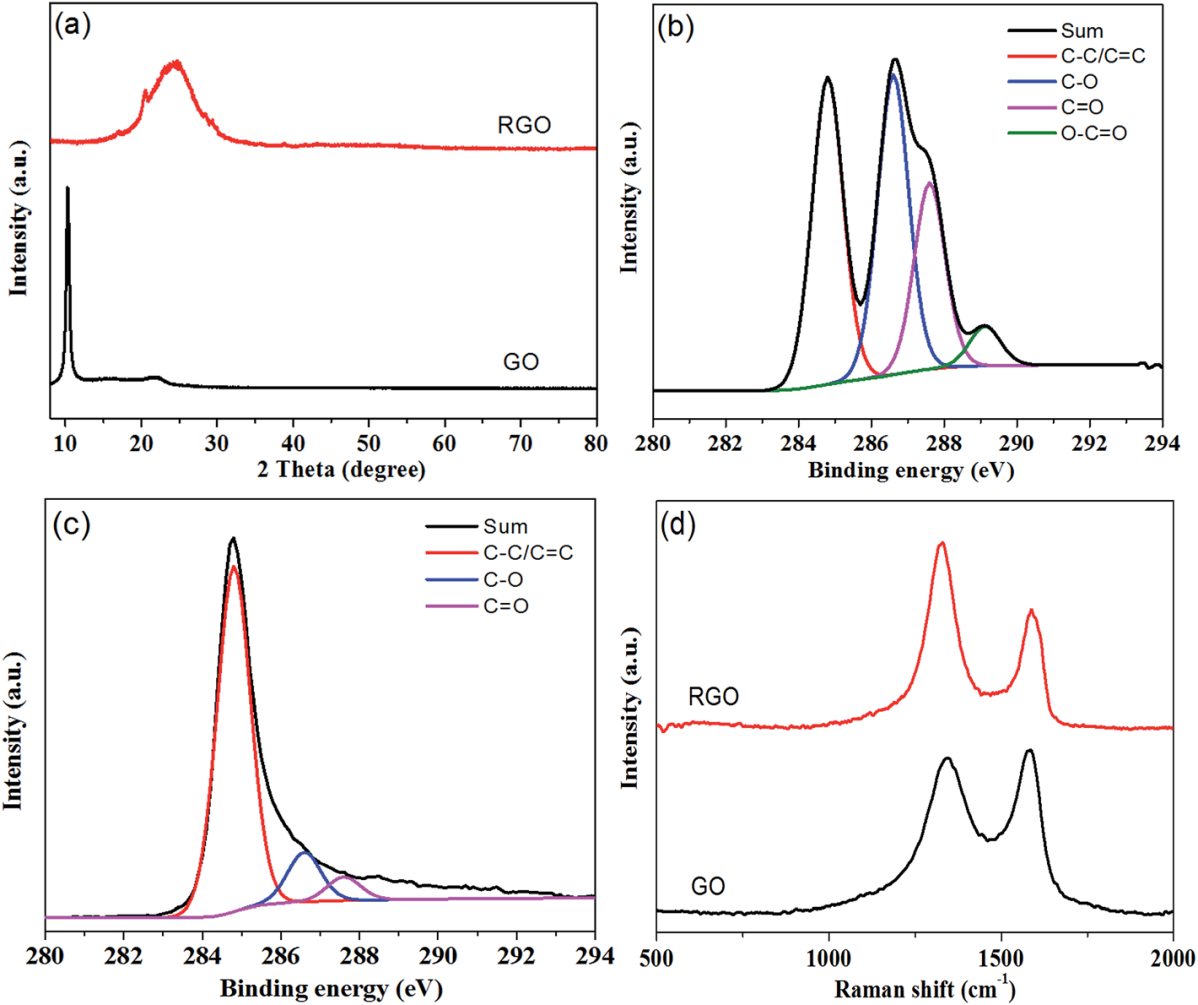
(a) XRD patterns of GO and RGO. (b) C 1s XPS spectra of GO. (c) C 1s XPS spectra of RGO. (d) Raman spectra of GO and RGO.

The morphology and structure of the PZT ceramics used were observed using SEM and XRD as shown in Fig. S1(a, b and c)†. The results showed that the piezoelectric ceramics possess polycrystallized perovskite structures with an average size of approximately 2–10 μm.^27–29^ As shown in Fig. 1, hydrazine hydrate was used to reduce GO, and after hydrothermal reduction, RGO sheets self-assembled spontaneously onto the skeleton of the polyurethane sponge to form PGF. The reduction process was proved by the XRD patterns, XPS and Raman spectra. As shown in Fig. 2(a), the XRD pattern of GO exhibits a feature diffraction peak at about 10.3°. For RGO, this peak disappears and a relatively broad peak at about 25° is observed, which indicates that GO has been transformed into reduced graphene oxide with significantly less functionality.^30,31^Fig. 2(b) shows the C 1s XPS spectra of GO, and four different peaks centered at 284.8, 286.6, 287.6 and 289.1 eV corresponding to C=C/C–C, C–O, C=O and O–C=O, respectively, are observed. For RGO, the intensity of the peaks corresponding to oxygen-containing groups decrease dramatically, especially for the C–O peak, as shown in Fig. 2(c), demonstrating a considerable reduction of GO.^32^ The results confirm the removal of oxygen groups after reduction and indicate that the delocalized π conjugation is restored in our RGO sample. The Raman spectra of GO and RGO are shown in Fig. 2(d). The peak centered at 1346 cm^−1^ is assigned to the D band, which is associated with structural imperfections caused by the defects and functional groups. The peak located at 1576 cm^−1^ is attributed to the G band, which is characteristic of the sp^2^-hybridized carbon–carbon bonds.^33^ The peak area ratio of the D band to the G band for RGO is increased from 1.58 to 2.10 when compared with that of GO. According to previous reports, the increase in the ratio of *A*(D)/*A*(G) indicates that more numerous but smaller sp^2^ carbon domains have partially recovered after the reduction.^34^ The results obtained from the XRD, XPS and Raman spectroscopy strongly suggest that GO is effectively reduced to RGO by hydrazine hydrate.

**Fig. 3:**
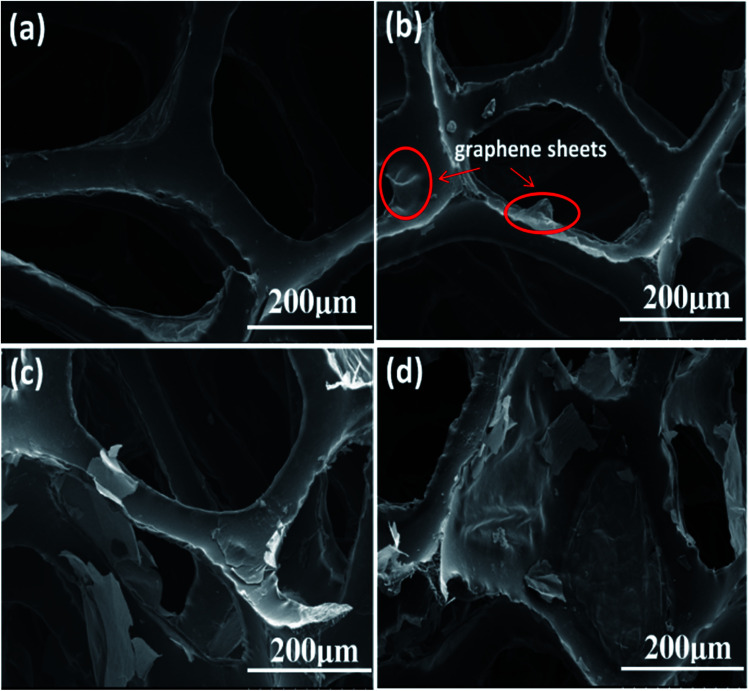
SEM images of (a) polyurethane foam, (b) 0.1 mg ml^−1^ PGF (PGF-0.1), (c) 0.5 mg ml^−1^ PGF (PGF-0.5) and (d) 1 mg ml^−1^ PGF (PGF-1).

As mentioned above, the volume resistivity of the piezo-damping composites should be adjusted to be in the semiconductor range, at approximately 10^6^–10^8^ Ω cm as reported in other works.^21,22^ PGFs with different amounts of RGO sheets were prepared to confirm the proper dosage of the conductive phase. The morphology and structure of the polyurethane foam and the fabricated PGFs with different RGO amounts were observed using SEM, as shown in Fig. 3. It was found that both the PU template and the prepared PGFs possess a three-dimensional highly porous structure with a uniform pore size of approximately several hundred micrometers. The RGO sheets can be attached onto the skeleton of the PU foam during the hydrothermal reduction process due to their hydrophobicity and π–π complexation interactions. From the SEM pictures of the sample PGF-0.1 (Fig. 3(b)), it can be seen that the RGO sheets have a scattered distribution on the skeleton of the PU foam. For the sample PGF-0.5, the skeleton of the PU foam is mostly covered with RGO sheets and some pores can also be blocked by some RGO sheets (Fig. 3(c)). For the sample PGF-1, as the RGO content continues to increase, most of the pores of the PU template are covered with RGO sheets and more RGO sheets stack on the skeletons, as shown in Fig. 3(d). With increasing amounts of RGO, the electrical conductivity of the PGFs will be correspondingly improved.

**Fig. 4:**
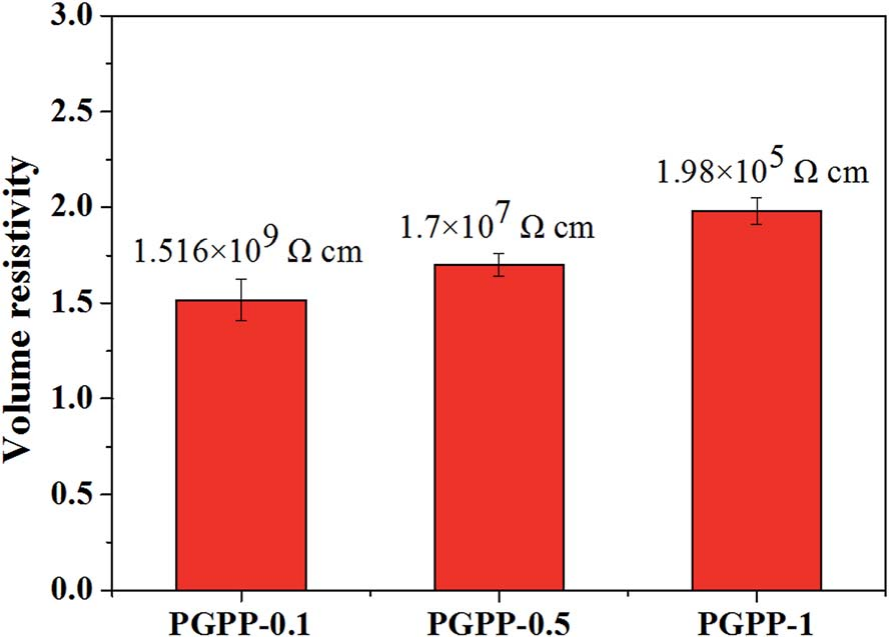
The volume resistivity values of PGPP composites with different RGO amounts (mass ratio of PZT/PDMS held constant at 1 : 1).

PGPP composites with different RGO levels were fabricated by filling the above PGFs with a PZT and PDMS mixture (mass ratio of PZT/PDMS equaling 1 : 1), and their volume resistivity values were measured to find out the suitable RGO loading. A variation plot of the volume resistivity values of PGPPs with different RGO amounts is shown in Fig. 4. It can be observed that the volume resistivity of the fabricated PGPP composites decreases with increasing RGO content, showing values of 1.52 × 10^9^, 1.7 × 10^7^ and 1.98 × 10^5^ Ω cm for the samples PGF-0.1, PGF-0.5 and PGF-1, respectively. As described in other works, the conductivity of a piezo-damping composite being too high or too low are both disadvantageous for the dissipation of external mechanical energy,^18,19,21,22^ and thus PGF-0.5 was chosen as the conductive network of the PGPP composites in the following study. Moreover, the calculated mass ratio of RGO/PDMS approximately equalled 0.05 wt%, which is dramatically decreased when compared with previous reports.^21–25^ This is beneficial for both reducing the cost and making the fabricated PGPPs retain the flexible behavior of the PDMS matrix.

**Fig. 5:**
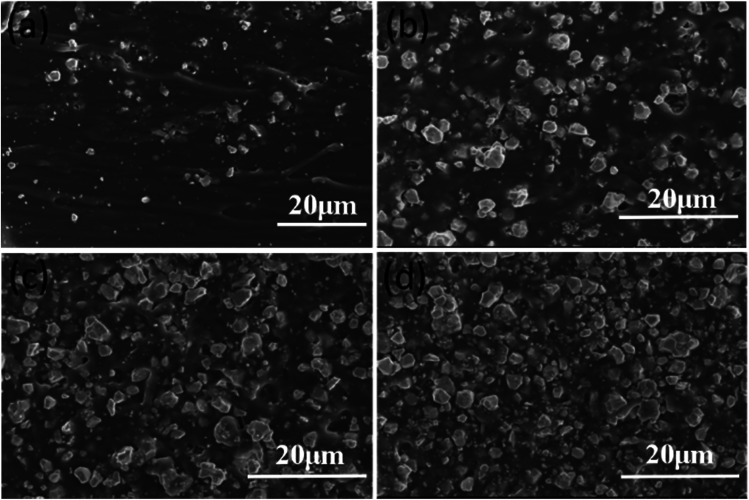
SEM images of the PGPP composites with different amounts of PZT ceramics: mass ratio of PZT/PDMS equaling (a) 1 : 1, (b) 2 : 1, (c) 4 : 1 and (d) 6 : 1.

Using PGF-0.5 as the conductive network, composite PGPPs with different PZT amounts were prepared, and their SEM images are shown in Fig. 5. It was found that the PZT ceramics are dispersed uniformly in the PDMS matrix and they exhibit good wetting with the polymer for all samples. Moreover, with an increased amount of PZT, the piezoelectric ceramics gradually become the major component of the PGPP composites, which is beneficial for external energy dissipation *via* the piezo-damping effect. When the mass ratio of PZT and PDMS is further increased to 8 : 1, the viscosity of the mixture becomes too high, and consequently there are too many bubbles left in the composites after the curing reaction, which exerts a negative effect on the mechanical and damping properties of the PGPP composite, therefore it was not studied any further.

**Fig. 6:**
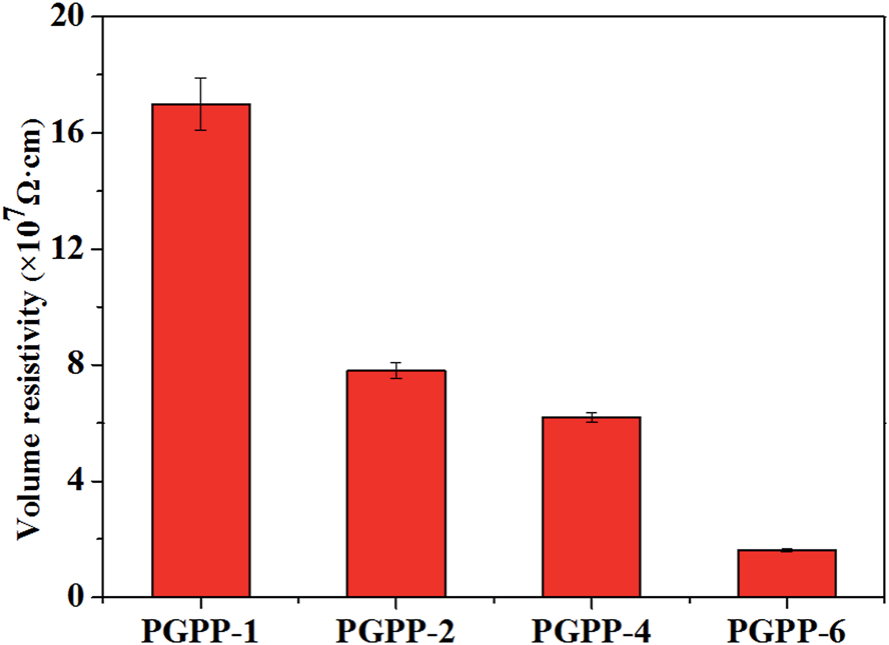
A variation plot of the volume resistivity of the PGPP composites with different loadings of the PZT ceramics.

**Fig. 7:**
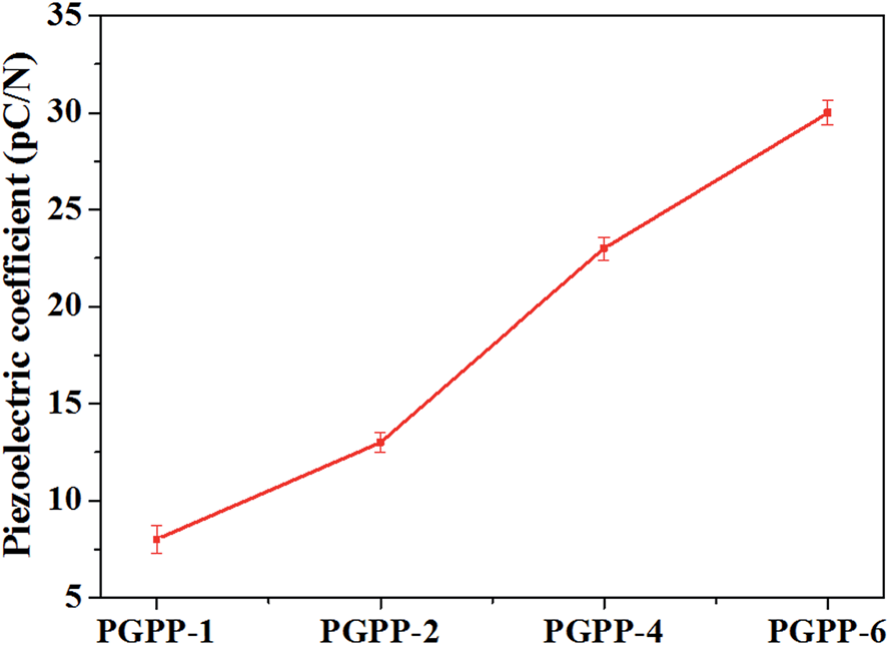
A variation plot of the piezoelectric coefficient (*d*_33_) values of the PGPP composites with various PZT levels.

The volume resistivity values of the PGPP composites with different PZT amounts were measured and the results are shown in Fig. 6. It can be seen that the volume resistivity (*R*_v_) values for PGPP-1, PGPP-2, PGPP-4 and PGPP-6 are 17 × 10^7^, 7.8 × 10^7^, 6.2 × 10^7^ and 1.6 × 10^7^ Ω cm, respectively, which are all adjusted in the semiconductor range and favourable for the function of the piezo-damping effect. Moreover, as the content of PZT ceramics was increased, the *R*_v_ values gradually decreased, which is a similar result to that in previous work.^22,24^ In addition, the piezoelectric coefficient (*d*_33_) values of the fabricated PGPP composites were measured, and the results are shown in Fig. 7. The results show that the piezoelectric coefficient increases with increased PZT loading, and the *d*_33_ values of the composites PGPP-1, PGPP-2, PGPP-4 and PGPP-6 are 8, 13, 23 and 30 pC N^−1^, respectively.

**Fig. 8:**
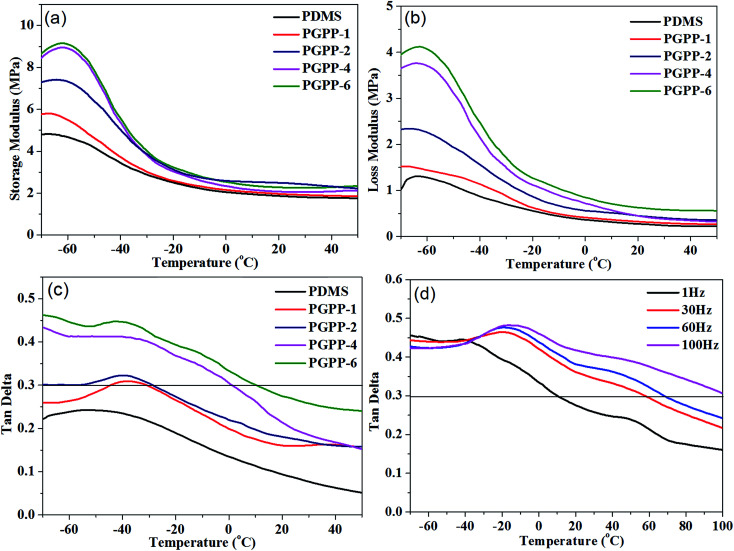
The variation plots of (a) storage modulus, (b) loss modulus and (c) loss factor as a function of temperature for the PDMS matrix and the fabricated piezo-damping PGPP composites at 1 Hz. (d) The variation curves of loss factor as a function of temperature for PGPP-6 at different frequencies.

The damping properties of the PDMS matrix and the fabricated PGPP composites were investigated using a Dynamic Mechanical Analyzer (DMA), and the parameters of storage modulus (*E*′), loss modulus (*E*′′) and loss factor (tan *δ*) of the materials were obtained. Fig. 8(a) shows the PDMS and PGPPs’ *E*′ values with different PZT loadings as a function of temperature. The storage modulus is an important property that is used to assess the load bearing capacity of a material, and a high *E*′ value means a high stiffness of the material.^35^ It was found that all of the fabricated PGPP composites exhibit an increased storage modulus compared with the PDMS matrix, and as the content of PZT ceramics is increased, the *E*′ values of the PGPPs improve correspondingly. Table 1 shows that the maximum *E*′ values of the PDMS matrix, PGPP-1, PGPP-2, PGPP-4 and PGPP-6 are 4.82, 5.80, 7.41, 8.95 and 9.15 MPa, respectively, which indicates that the PGPPs possess increased mechanical properties compared to the PDMS matrix. This may be due to the addition of high modulus PZT ceramics and graphene sheets. The composite PGPP-6 exhibits the best storage modulus behavior and its *E*′ value represents an increase of about 89.8% compared to the PDMS matrix.

**Table 1:** The influence of different PZT loadings on the damping behavior of PGPPs at 1 Hz from −70 to 50 °C

Sample	Storage modulus (*E*′) (MPa)	Loss modulus (*E*′′) (MPa)	Loss factor (tan *δ*) at *T*_g_	*T*_g_ (°C)	Temperature range (°C)/tan *δ* > 0.3 (Δ*T*)
PDMS	4.82	1.31	0.24	−51.2	0
PGPP-1	5.80	1.52	0.31	−38.1	−44.1 to −31.5 (12.6)
PGPP-2	7.41	2.34	0.32	−39.7	−70 to −29.1 (40.9)
PGPP-4	8.95	3.76	0.41	−45	−70 to 0.4 (70.4)
PGPP-6	9.15	4.12	0.45	−41.2	−70 to 9.8 (79.8)

Fig. 8(b) shows the loss modulus values of the PDMS matrix and the fabricated PGPP composites as a function of temperature. The loss modulus (*E*′′) is a measure of the energy dissipated as heat per unit cycle under mechanical deformation and it is used to characterize the viscosity of a material.^6,7^ It is evident that all of the fabricated PGPPs show higher *E*′′ values than the PDMS matrix, which indicates that the PGPP composites could dissipate more mechanical vibration and noise as heat energy. Moreover, the loss modulus values of the PGPPs improve correspondingly with an increase of the PZT content. As shown in Table 1, the maximum *E*′′ values for PDMS, PGPP-1, PGPP-2, PGPP-4 and PGPP-6 are 1.31, 1.52, 2.34, 3.76 and 4.12 MPa, respectively. The composite PGPP-6 possesses the best loss modulus behavior and its *E*′′ value shows an increase of about 214.5% compared to the PDMS matrix.

The loss factor is defined as the ratio of storage modulus to loss modulus and a higher tan *δ* value indicates the better energy dissipation capability of a material. Normally, the tan *δ* value of an engineering damping material is required to be higher than 0.3 and the temperature range where tan *δ* > 0.3 should be as wide as possible.^8,9,25^ The loss factor values of the PDMS matrix and PGPP composites as a function of temperature are shown in Fig. 8(c). It is evident that the loss factor values of all piezo-damping PGPP composites have improved greatly compared with that of the PDMS matrix over the whole measured temperature range of −70 to 50 °C. The energy dissipation routes of the PGPP composite are mainly the piezo-damping effect, the friction between filler–filler and filler–matrix, and the viscoelasticity of the polymer matrix. When a PGPP composite is subjected to an external alternating force, some mechanical energy is transformed into electrical energy *via* the piezoelectric effect of the PZT ceramics, and then the generated electricity is dissipated as heat when flowing through the PGF semiconductor network.^19,21–23^ In addition, under an external alternating force, the PGPP composites undergo a certain deformation, which can cause boundary sliding (filler–filler) and interfacial sliding (filler–matrix), thus dissipating some mechanical energy.^4,8,11,13^ Moreover, friction caused by the local movement of macromolecule chains of the polymer matrix near *T*_g_ can dissipate most of the mechanical energy as heat. As shown in Table 1, the maximum tan *δ* value for PGPP-6 is 0.45, which is improved by about 87.5% compared to the PDMS matrix, and the temperature where tan *δ* > 0.3 is broadened to −70 to 9.8 °C, demonstrating that PGPP-6 can be used as good engineering damping material.

Since frequency has a direct impact on the mobility of macromolecule chains, which is directly related to the damping properties of polymers,^8,15^ the damping performance of the composite PGPP-6 under different frequencies in the temperature range of −70 to 100 °C was studied and the results are shown in Fig. 8(d). It was found that for the composite PGPP-6, the glass transition temperature and the loss factor were enhanced correspondingly with increasing frequency. According to the time–temperature equivalence principle of polymers, for a relaxation process, temperature and time are inversely related, which means that high temperature is equivalent to a short time (or high frequency) and *vice versa*. According to the theory, the *T*_g_ of polymers can be seen at high temperature and high frequency, and can also be observed at low temperature and low frequency, therefore the *T*_g_ of the composite PGPP-6 shifts to higher temperature as the frequency increases. Moreover, the loss factor of PGPP-6 is enhanced correspondingly with increasing frequency. For PGPP-6 at 30 Hz, the temperature range where tan *δ* > 0.3 is −70 to 57 °C, which could cover the working temperature range of most engineering materials, and thus PGPP-6 can be used as a good damping material in a wide temperature range under different frequencies (Table 2).

**Table 2:** Influence of different frequencies on the damping behavior of PGPP-6 from −70 to 100 °C

Frequency (Hz)	Loss factor (tan *δ*) at *T*_g_	*T*_g_ (°C)	Temperature range (°C)/tan *δ* > 0.3 (Δ*T*)
1	0.45	−41.2	−70 to 9.8 (79.8)
30	0.46	−19.8	−70 to 57 (127)
60	0.47	−18.6	−70 to 68 (138)
100	0.48	−16.2	−70 to 100 (170)

## 4. Conclusion

In this paper, a novel piezo-damping polyurethane-based graphene foam (PGF)/PZT/PDMS composite (PGPP) was facilely fabricated using a one-step vacuum-assisted filling method. Using three-dimensional PGF as the conductive phase, the content of RGO sheets at the percolation threshold is only 0.05 wt%, which shows an obvious reduction compared with other reports, and it can also easily guarantee the uniform distribution of RGO sheets in the composites. The storage modulus of the PGPP-6 composite is increased by approximately 89.8% compared with the PDMS matrix due to the addition of evenly dispersed high modulus PZT ceramics and RGO sheets. In addition, the loss factor of PGPP-6 improves by about 87.5% compared to the polymer matrix due to the external mechanical energy to electrical energy to heat piezo-damping effect and the friction effect. Furthermore, the damping behavior of PGPP-6 can be enhanced greatly with increasing frequency, and its temperature range where tan *δ* > 0.3 is −70 to 57 °C at 30 Hz, which covers the usual applied temperature range of engineering damping materials. Since the fabricated PGPPs possess good flexibility, they can be used as good surface coating damping materials in a wide temperature range at different frequencies.

## Conflicts of interest

There are no conflicts to declare.

## Supplementary Material

RA-008-C8RA00266E-s001

## References

[cit1] Vinogradov A. M., Schmidt V. H., Tuthill G. F., Bohannan G. W. (2004). Mech. Mater..

[cit2] Rajoria H., Jalili N. (2005). Compos. Sci. Technol..

[cit3] Finegan I. C., Gibson R. F. (1999). Compos. Struct..

[cit4] Chandra R., Singh S. P., Gupta K. (1999). Compos. Struct..

[cit5] Khashaba U. A. (2015). Composites, Part A.

[cit6] Wang J., Liu R., Li W., Li Y., Tang X. (2015). Polym. Int..

[cit7] Yu X., Gao G., Wang J., Li F., Tang X. (2015). Polym. Int..

[cit8] Qin C.-L., Cai W.-M., Cai J., Tang D.-Y., Zhang J.-S., Qin M. (2004). Mater. Chem. Phys..

[cit9] Lv X., Huang Z., Shi M., Fan Y., Gao G. (2016). RSC Adv..

[cit10] Chem Y. C., Tseng S. M., Hsieh K. H. (2015). J. Appl. Polym. Sci..

[cit11] Wang T., Chen S., Wang Q., Pei X. (2010). Mater. Des..

[cit12] Wang C., Jia J. (2014). High Perform. Polym..

[cit13] Chen S., Wang Q., Wang T., Pei X. (2011). Mater. Des..

[cit14] Chen S., Wang T., Wang Q. (2016). J. Polym. Mater..

[cit15] Shih Y.-F., Jeng R.-J. (2002). J. Appl. Polym. Sci..

[cit16] Tanimoto T. (2007). Compos. Sci. Technol..

[cit17] Alipour Skandani A., Masghouni N., Case S. W., Leo D. J., Al-Haik M. (2012). Appl. Phys. Lett..

[cit18] Carponcin D., Dantras E., Michon G., Dandurand J., Aridon G., Levallois F., Cadiergues L., Lacabanne C. (2015). J. Non-Cryst. Solids.

[cit19] Zhang C., Sheng J. F., Ma C. A., Sumita M. (2005). Mater. Lett..

[cit20] Sharma S. K., Gaur H., Kulkarni M., Patil G., Bhattacharya B., Sharma A. (2013). Compos. Sci. Technol..

[cit21] Hori M., Aoki T., Ohira Y., Yano S. (2001). Composites, Part A.

[cit22] Tian S., Cui F., Wang X. (2008). Mater. Lett..

[cit23] Sumita M., Gohda H., Asai S., Miyasaka K. (1991). Makromol. Chem., Rapid Commun..

[cit24] Wang Y., Yan H., Huang Z., Zhang T. (2012). Polym.-Plast. Technol. Eng..

[cit25] Liu Z., Wang Y., Huang G., Wu J. (2008). J. Appl. Polym. Sci..

[cit26] Gilje S., Han S., Wang M., Wang K. L., Kaner R. B. (2007). Nano Lett..

[cit27] Lin Y., Liu Y., Sodano H. A. (2009). Appl. Phys. Lett..

[cit28] Park K., Son J. H., Hwang G.-T., Jeong C. K., Ryu J., Koo M., Choi I., Lee S. H., Byun M., Wang Z. L., Lee K. J. (2014). Adv. Mater..

[cit29] Xu G., Jiang W., Qian M., Chen X., Li Z., Han G. (2009). Cryst. Growth Des..

[cit30] Moon I. K., Lee J., Ruoff R. S., Lee H. (2010). Nat. Commun..

[cit31] Compton O. C., Dikin D. A., Putz K. W., Brinson L. C., Nguyen S. T. (2010). Adv. Mater..

[cit32] Zhang X., Sui Z., Xu B., Yue S., Luo Y., Zhan W., Liu B. (2011). J. Mater. Chem..

[cit33] Gupta A., Chen G., Joshi P., Tadigadapa S., Eklund P. C. (2006). Nano Lett..

[cit34] Zhang X.-J., Wang G.-S., Cao W.-Q., Wei Y.-Z., Cao M.-S., Guo L. (2014). RSC Adv..

[cit35] Zhang C., Li H., Zhuo Z., Dugnani R., Xue W., Zhou Y., Chen Y., Liu H. (2017). RSC Adv..

