# Supplementary material for: Facile fabrication of polyurethane-based graphene foam/lead zirconate titanate/polydimethylsiloxane composites with good damping performance
Source: RSC Adv. 2018 Feb 20;8(15):7916–23. doi: 10.1039/c8ra00266e (PMC9078480; doi:10.1039/c8ra00266e)
Supplement: RA-008-C8RA00266E-s001 [file RA-008-C8RA00266E-s001.pdf]

Electronic Supplementary Material (ESI) for RSC Advances.
This journal is © The Royal Society of Chemistry 2018

Supporting Information

Facile fabrication of polyurethane-based graphene foam/lead zirconate titanate/polydimethylsiloxane composites with good damping performance

Chunmei Zhang[a,1], Yujie Chen[a,1], Hua Li[a,b,*], Wenchao Xue[a], Ran Tian[a], Roberto Dugnani[c], Hezhou Liu[a,b]

[a]State Key Laboratory of Metal Matrix Composites, School of Materials Science and Engineering, Shanghai Jiao Tong University, Dongchuan Road No. 800, Shanghai 200240, China.

[b]Collaborative Innovation Center for Advanced Ship and deep-Sea Exploration, Shanghai Jiao Tong University.

[c]Michigan-Shanghai Jiao Tong University Joint Institute, China.

[*]Corresponding author: Hua Li (email: lih@sjtu.edu.cn)

[1]These authors contributed equally to this work.

Figure S1 The SEM images (a) and (b) and the XRD spectra (c) of the PZT ceramics used
